# Clinical Applications of Quantitative Perfusion Imaging with a C-Arm Flat-Panel Detector—A Systematic Review

**DOI:** 10.3390/diagnostics13010128

**Published:** 2022-12-30

**Authors:** Abdallah H. A. Zaid Al-Kaylani, Richte C. L. Schuurmann, Wouter D. Maathuis, Riemer H. J. A. Slart, Jean-Paul P. M. De Vries, Reinoud P. H. Bokkers

**Affiliations:** 1 Department of Radiology, Medical Imaging Center, University Medical Center Groningen, University of Groningen, 9712 CP Groningen, The Netherlands; 2 Department of Surgery, Division of Vascular Surgery, University Medical Center Groningen, University of Groningen, 9712 CP Groningen, The Netherlands; 3 Department of Biomedical Photonic Imaging, Faculty of Science and Technology, University of Twente, 7522 NB Enschede, The Netherlands; 4 Department of Nuclear Medicine & Molecular Imaging, Medical Imaging Center, University of Groningen, 9712 CP Groningen, The Netherlands

**Keywords:** C-arm, flat-panel detector, cone-beam computed tomography, perfusion imaging, perfusion angiography, dual-phase perfusion

## Abstract

C-arm systems with digital flat-panel detectors are used in interventional radiology and hybrid operating rooms for visualizing and performing interventions on three-dimensional structures. Advances in C-arm technology have enabled intraoperative quantitative perfusion imaging with these scanners. This systematic review provides an overview of flat-panel detector C-arm techniques for quantifying perfusion, their clinical applications, and their validation. A systematic search was performed for articles published between January 2000 and October 2022 in which a flat-panel detector C-arm technique for quantifying perfusion was compared with a reference technique. Nine articles were retrieved describing two techniques: two-dimensional perfusion angiography (n = 5) and dual-phase cone beam computed tomography perfusion (n = 4). A quality assessment revealed no concerns about the applicability of the studies. The risk of bias was relatively high for the index and reference tests. Both techniques demonstrated potential for clinical application; however, weak-to-moderate correlations were reported between them and the reference techniques. In conclusion, both techniques could add new possibilities to treatment planning and follow-up; however, the available literature is relatively scarce and heterogeneous. Larger-scale randomized prospective studies focusing on clinical outcomes and standardization are required for the full understanding and clinical implementation of these techniques.

## 1. Introduction

Interventional radiology suites have traditionally used two-dimensional (2D) techniques such as digital subtraction angiography (DSA) for guiding operations and intervening on three-dimensional (3D) structures [1]. DSA is a fluoroscopic technique that enables the visualization of small vessels with small amounts of contrast through the means of image subtraction [2]. Advances in technology allowed the development of rotational DSA in which 3D volumes can be acquired in a single gantry rotation using cone-beam backprojection algorithms [3,4,5].

Cone-beam computed tomography (CBCT) technology was incorporated in the late 1990s into experimental C-arm computed tomography (CT) systems using image intensifier systems based on a convolution-backprojection formula [1,6]. These image intensifier systems were later replaced with digital flat-panel detectors, which improved spatial and contrast resolution [7]. Nowadays, flat-panel detector C-arm systems are widely used in hybrid operating rooms and interventional radiology suites for treatment planning, intraoperative guidance, and intra-arterial therapy.

Most pathologies encountered in the interventional suite and their targeted therapies have a direct effect on the dynamics of blood flow, raising the demand for a standardized and quantitative evaluation of tissue perfusion. In the diagnosis and treatment of vascular diseases, 2D DSA is the most often used imaging method [8,9,10]. Studies have demonstrated the feasibility of extracting quantitative perfusion data from 2D DSA through the means of parametric color coding [11,12,13,14,15,16,17] and time–density curve (TDC) analysis [18,19,20]. Furthermore, in patients with cerebral ischemia and hepatic malignancies, studies have investigated 3D volumetric imaging with flat-panel detector C-arm systems for quantifying perfusion using techniques such as multiphase CBCT perfusion [21] and dual-phase CBCT perfusion [22].

Quantitative perfusion imaging with C-arm systems is applied across a varied spectrum of diseases, from the detection of perfusion deficits in acute ischemic stroke to the treatment of liver tumors and peripheral arterial disease (PAD). The aim of this systematic review is to provide an overview of flat-panel detector C-arm techniques for quantifying perfusion, their clinical applications, and their validation against reference techniques.

## 2. Materials and Methods

This systematic review was conducted in accordance with the Preferred Reporting Items for Systematic Review and Meta-Analysis (PRISMA) statement [23].

### 2.1. Literature Search

PubMed, EMBASE, Cumulative Index of Nursing and Allied Health (CINAHL), and the Cochrane library were searched for eligible studies on perfusion imaging techniques using a flat-panel detector C-arm. The search used free text, Medical Subject Headings terms, and Boolean operators. Additional studies were identified by cross-reference checks of the relevant literature. The complete search strategy is available in Appendix A. The search was limited to studies published between January 2000 and October 2022 because the technology in studies published before January 2000 is considered outdated.

The initial screening of the titles and abstracts of the studies was conducted by two authors (A.H.A.Z.A.-K., W.D.M.), independent of each other, by means of Rayyan software (Rayyan Systems Inc., Cambridge, MA, USA) [24]. Both reviewers (A.H.A.Z.A.-K., W.D.M.) were blinded to the journal titles and authors. Disagreement was discussed by the two reviewers to reach consensus in inclusion. A third reviewer (R.B.) provided adjudication when consensus could not be reached.

### 2.2. Selection Criteria

Articles including a perfusion imaging technique using a flat-panel detector C-arm were eligible. The technique had to be compared against a reference technique using another method. The selected articles included human subjects only and were not limited to one language. Studies in languages other than English were translated using online translation services. Exclusion criteria were studies investigating intracranial and/or cardiac pathologies, case reports, case-series studies, studies with fewer than 10 subjects, studies without full text availability, commentaries, letters to the editors, conference abstracts, and review articles. Cranial and cardiac pathologies were considered beyond the scope of this review and were excluded due to the large heterogeneity between the studies.

### 2.3. Data Extraction and Quality Assessment

A predefined form was used for the extraction of study details, which were categorized according to the technique for measuring tissue perfusion. The following data were extracted: study year, study design, number of patients, diagnoses, C-arm technique, reference technique, and the measured perfusion parameters and the correlations between them. Rayyan software was used for data collection [24]. The quality and risk of bias of the included articles were appraised with the Quality Assessment of Diagnostic Accuracy Studies (QUADAS-2) tool by one reviewer (A.Z.A.-K) [25].

## 3. Results

**Figure 1:**
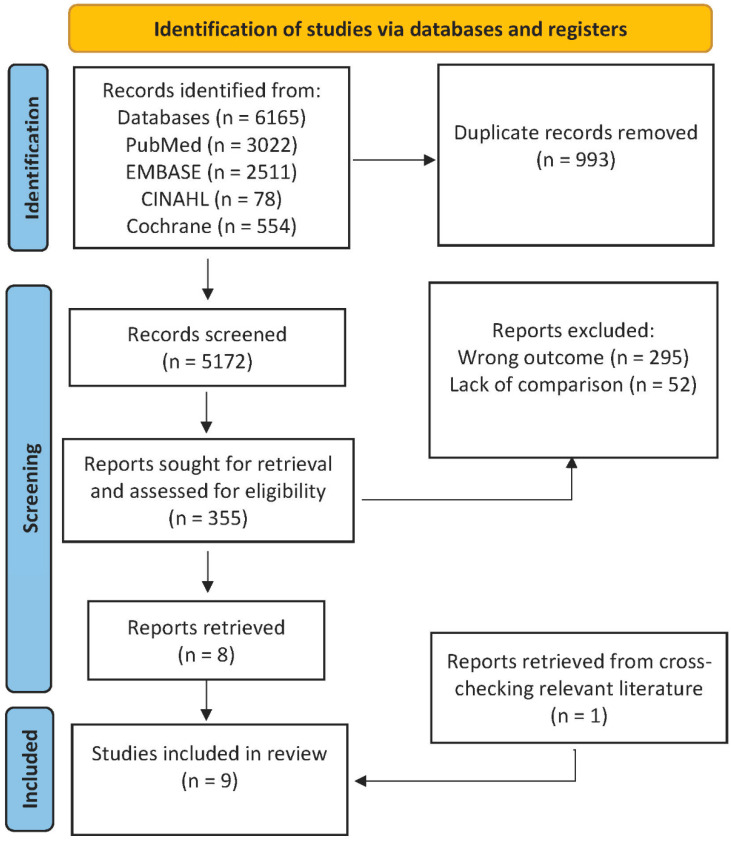
Diagram of the selection process.

We retrieved 6165 studies, of which 993 were duplicates. After an initial evaluation of the titles and abstracts was conducted, 355 articles were selected for full-text assessment. Of these 355 articles, 295 were excluded because of different outcomes and 52 were excluded because no comparison was available to a reference technique. One article was retrieved from cross-checking the references in the relevant literature. Nine articles were eligible for inclusion. The review process is outlined in Figure 1.

**Figure 2:**
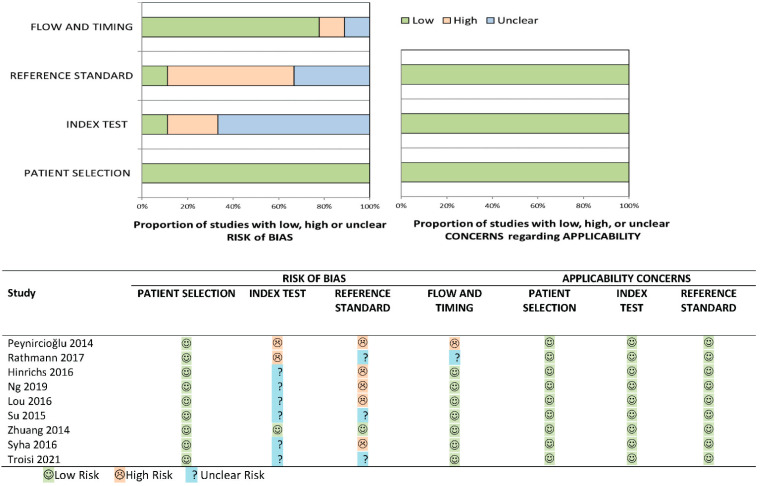
Bias and quality assessment of the included studies according to QUADAS-2.

Five articles in this review investigated 2D perfusion angiography in patients with peripheral arterial disease (PAD) undergoing endovascular therapy. The remaining four studies investigated dual-phase CBCT perfusion imaging in patients with hepatocellular carcinoma (HCC). Table 1 provides an overview of the studies. Figure 2 shows the quality and risk of bias assessment of the included studies. Little to no concerns were found for the applicability and risk of bias in patient selection and flow and timing. However, the risk of bias was relatively high in most of the studies for the index and reference tests.

### 3.1. 2D Perfusion Angiography

#### 3.1.1. Description of the Technique

DSA images consist of contrast-enhanced vascular structures free of unwanted background details through means of image subtraction and enhancement [35,36,37,38,39]. These processes occur in real time, meaning they are rapid enough to allow live visualization of structures with the ability to interact during the operational procedure. However, these images only provide visual qualitative perfusion data, without any quantification.

2D perfusion angiography is a novel technique in which parametric color coding and TDC analysis are applied to available conventional DSA images for generating quantitative perfusion data. With parametric color coding, the time delay between contrast injection and peak contrast density is defined for each pixel and converted into colors ranging from the shortest (red) to the longest (blue) time delay. Furthermore, with 2D perfusion angiography, the concentration of intravenously injected contrast in a region of interest (ROI) in a DSA series is visualized and monitored over time for the generation of the TDCs of the bolus [18,19,20]. Two-dimensional perfusion angiography is performed intraoperatively within seconds via postprocessing, without requiring extra contrast or radiation.

The technique was first described by Jens et al. [18]. The standardization of image acquisition and contrast injection is necessary to ensure the reliability of the results. Furthermore, 2D perfusion angiography is sensitive to motion artifacts, necessitating the fixation of the foot and leg [18,19,20,40]. A lateral projection is performed before and after the procedure. For the injection protocol, iodixanol (320 mg iodine/mL; Visipaque; GE Healthcare, Eindhoven, the Netherlands) is injected with an infusion pump at a rate of 3 mL/s. DSA acquisition is started simultaneously with pump injection at a rate of 3 frames/s.

Perfusion analysis and matching of the pre- and postprocedural ROIs are run with vendor-specific postprocessing software. From this analysis, a number of perfusion parameters are calculated: arrival time of contrast, time to peak (TTP), peak density (PD), TDC of the bolus, and area under the (time–density) curve (AUC). TTP reflects the time from the beginning of acquisition to maximal tissue density within a selected ROI; PD marks the maximal tissue density during acquisition; and the AUC refers to the density values during the entirety of acquisition in a selected ROI [20].

#### 3.1.2. Clinical Applications

Three retrospective and two prospective studies were selected that investigated the use of 2D perfusion angiography. All studies were performed in patients with PAD undergoing endovascular therapy. A detailed overview of the studies is provided in Table 1.

Lou et al. [28] retrospectively compared change (Δ) in the TTP (ΔTTP) derived by 2D perfusion angiography against change in the ankle–brachial index (ΔABI) in 19 patients. The proximal superficial femoral artery was selected as a reference for the TTP. The TTP was defined as the interval of time between medial and lateral plantar/dorsalis pedis relative to the proximal superficial femoral artery. A good correlation (r = 0.86) between the ΔTTP and ΔABI was reported.

Ng et al. [27] retrospectively compared 2D perfusion angiography against changes in the ABI and/or toe–brachial index in 47 patients. From the generated TDCs, the washout gradient was derived and defined as the average contrast flow velocity before reaching maximum contrast intensity. The washout gradient was further quantified by deriving additional parameters, namely, the percentage of contrast decay after the peak (at 1, 2, 3, 4, and 5 s) and the time required for contrast intensity to decay after peak intensity to a certain percentage (50%, 60%, 70%, 80%, and 90%). The changes in the AUC and the washout parameters were correlated to changes in the ABI or toe–brachial index. For both contrast decay percentage and decay time, the reported correlations (*r* = 0.34–0.48 and *r* = 0.29–0.32, respectively) were weak. For the AUC, no significant correlation was found.

Su et al. [29] prospectively compared the ΔTTP derived from 2D perfusion angiography against the ΔABI and Δ transcutaneous oxygen pressure in 21 patients. Local (tibiofibular arteries) and regional (ankle) TTP values were derived from the TDCs. For the Δ local TTP, the correlations with the ΔABI and Δtranscutaneous oxygen pressure (*r* = 0.65 and *r* = 0.73, respectively) were moderate. For the Δ regional TTP, the correlations with the ΔABI and Δtranscutaneous oxygen pressure (*r* = 0.60 and *r* = 0.60, respectively) were good to moderate.

Hinrichs et al. [26] retrospectively compared 2D perfusion angiography against the ABI in 21 patients. The technique was adjusted, however, and the standardized protocol was not used. Instead, ROIs were placed proximal and distal to the vascular obstruction to assess arterial input and output, respectively. Taking into account the arterial input function, 2D perfusion angiography was conducted independently of pump injections. Ratios (inflow/outflow) for the TTP, PD, and AUC were calculated. The absolute values and pre- and postprocedural changes were compared against the ABI and ΔABI, respectively. No significant correlations were reported between the absolute values. For the changes, the correlation for the ΔTTP and ΔABI (*r* = −0.53, *p* = 0.008) was moderate. For the ΔAUC and ΔPD, no significant correlations with the ΔABI (*r* = 0.33, *p* = 0.11 and *r* = 0.39, *p* = 0.05, respectively) were reported.

Troisi et al. [30] prospectively investigated the relationship between TDCs derived from 2D perfusion angiography with transcutaneous oxygen pressure and 6-month wound healing. TDCs were quantified through the number of pixels. They reported a non-significant association (*r* = −0.24; *p* = 0.30) between TDCs and transcutaneous oxygen pressure values. Furthermore, they reported a marginal association (odds ratio = 2.60, confidence interval = 37.50–571.40, *p* = 0.04) between an increase in pixels in an ROI of more than 50% and a higher 6-month wound-healing rate.

### 3.2. Dual-Phase CBCT Perfusion

#### 3.2.1. Description of the Technique

Dual-phase CBCT perfusion is a 3D imaging technique for quantifying perfusion via generating perfusion maps of blood volume. With this technique, two 3D-rotational volumes of an ROI are acquired: a mask (baseline) volume and a fill (contrast-enhanced) volume. The acquisition of two 3D-rotational volumes of different contrast phases enables the extraction of absolute quantitative perfusion maps of blood volume (parenchymal blood volume (PBV)) in an ROI from the brain, liver, or hepatic tumors. Details regarding cerebral PBV were beyond the scope of the current review.

The measurement of liver PBV refers to the arterial blood volume of liver parenchyma and hepatic tumors at any moment, and is based on the assumption of a steady state of contrast media in the imaged liver and tumors during an acquisition run [22]; therefore, PBS is assumed to be constant during the time of acquisition [41]. Acquisition begins with a non-enhanced volume (mask image), after which the manual injection of contrast media is immediately initiated. An X-ray delay of a few seconds is used before the contrast-enhanced run (mask run) is begun to ensure that the contrast is in a steady state across the liver.

The postprocessing technique was first described by Zellerhoff et al. [22] and begins with the reconstruction and subtractions of the mask and fill runs. A non-rigid registration algorithm is used to correct the motion between the two runs. The steady-state arterial input function is automatically estimated from a histogram analysis of the vessels. Lastly, a final scaling and smoothing filter are applied.

#### 3.2.2. Clinical Applications

Three prospective and one retrospective study that investigated dual-phase CBCT perfusion imaging were found. All studies were performed in patients with HCC of the liver and compared CBCT against MDCT perfusion (MDCTP). An overview of the studies’ characteristics is provided in Table 1.

Zhuang et al. [31] prospectively investigated 20 patients with HCC before transarterial chemoembolization (TACE). Blood volume (BV) for the liver and tumors was extracted and compared between dual-phase CBCT perfusion and MDCTP. The correlations for the absolute values of tumor BV (*r* = 0.90, *p* < 0.001) and liver BV (*r* = 0.92, *p* < 0.001) were good.

Peynircioğlu et al. [32] prospectively investigated 10 patients with HCC and metastatic lesions before embolization. The BV values for 14 tumors were derived and the mean was calculated compared between dual-phase CBCT perfusion and MDCTP. A good correlation was reported (*r* = 0.97, *p* < 0.01).

Syha et al. [34] retrospectively compared changes in liver BV between dual-phase CBCT perfusion and MDCTP in 25 patients with HCC undergoing TACE. Three different estimation models were used for the estimation of liver BV from MDCTP: maximum slope [42], Patlak analysis [43], and deconvolution [44]. The overall correlations reported between tumor BV and the different BVs (maximum slope: *r* = 0.45, *p* = 0.005; Patlak analysis: *r* = 0.30, *p* = 0.02; and deconvolution: *r* = 0.24, *p* = 0.08) were weak. In smaller lesions (<3 cm), weak correlations were reported (maximum slope: *r* = 0.44, *p* = 0.01; Patlak analysis: *r* = 0.27, *p* = 0.14; and deconvolution: *r* = 0.26, *p* = 0.15). Moderate correlations were reported for lesions ≥ 3 cm (maximum slope: *r* = 0.60, *p* = 0.01; Patlak analysis: *r* = 0.54, *p* = 0.02; and deconvolution: *r* = 0.50, *p* = 0.04).

Rathmann et al. [33] prospectively investigated changes in liver BV derived by dual-phase CBCT perfusion in 16 patients with HCC undergoing TACE with drug-eluting beads. Changes in liver BV were correlated with changes in MDCTP parameters (arterial liver parenchyma, temporal maximum intensity projection, hepatic perfusion index, and portal venous parenchyma) and with the modified response evaluation criteria in solid tumors (mRECIST). The correlations reported (*r*^2^ = 0.06–0.23) were weak.

## 4. Discussion

This systematic review provides an overview of the currently available C-arm flat-panel detector imaging techniques for quantifying perfusion in extracranial and extracardiac vascular pathologies. Of the nine included studies in this review, five investigated 2D perfusion angiography in the lower limbs of patients with PAD, and the remaining four investigated dual-phase CBCT perfusion in the liver in patients with HCCs. Weak-to-moderate correlations were reported between the C-arm techniques and reference techniques. In addition, most of the studies included small cohorts and demonstrated a relatively high risk of bias in the conduct and interpretation of the index and reference tests.

Two-dimensional perfusion angiography provided an objective approach to the real-time quantification of the hemodynamic status of patients with PAD through means of parametric color coding and TDC analysis. The clinical results of revascularization therapies are highly unpredictable, as evident by high failure rates and repeat interventions [45,46,47]. Currently, DSA serves as the gold standard for the evaluation of treatment success in endovascular therapy, and is conducted through a subjective visual evaluation of the run-off of the affected vasculature [48,49]. However, the prediction of the clinical outcome after endovascular therapy remains challenging, with no clear objective endpoints [50]. Studies have investigated non-invasive measurements for the prediction of the clinical outcome; however, the techniques were limited by their unavailability intraoperatively and the lack of strong correlations with wound healing [50,51]. Direct correlations were reported between 2D perfusion angiography parameters and pressure indices and transcutaneous oxygen pressure. Furthermore, this technique has demonstrated excellent reliability when used with a standardized injection protocol [40] and has the potential to predict the clinical outcome [52]. However, there are some limitations, including the following: dependency on the acquisition protocol [40], sensitivity to foot movements [18,19,20,40], and sensitivity to inflammatory processes and arterial spasms [40]. In addition, owing to the heterogeneity in acquisition protocols and lack of clinical follow-up in the literature, no uniform endpoints for therapy have yet been determined.

The studies that applied 3D perfusion quantification techniques were in patients who underwent the hepatic arterial embolization of a liver tumor. The aim of these treatments is to maximize tumor devascularization, while causing minimal damage to liver parenchyma [53]. The primary endpoint of these therapies is overall survival [54,55]. No objective technique for the early prediction of the tumor response currently exists. Assessment is conducted postoperatively after one to three interventions via length measurements such as RECIST or mRECIST [56,57]. Therefore, enabling the early detection of the tumor response is valuable for assessment, follow-up, and treatment planning.

Interventional oncology under C-arm guidance is increasingly becoming a standard treatment in patients with liver tumors [58,59,60]. The advent of dynamic functional imaging with dual-phase CBCT perfusion has made intraoperative functional imaging of the liver possible, leading to new opportunities for the early assessment of the tumor response. Studies have demonstrated the feasibility of using dual-phase CBCT perfusion imaging for the calculation of the PBV of liver and hepatic tumors and validated it against MDCTP [31,32,33,34]. Zhuang et al. [31] and Peynircioğlu et al. [32] included small cohorts and reported good correlations, whereas Syha et al. [34] and Rathmann et al. [33] reported weak to moderate correlations. The discrepancy in the correlations suggests that dual-phase CBCT perfusion and MDCTP measurements could indicate independent changes [33]. Despite this discrepancy, dual-phase CBCT perfusion has been demonstrated to have a similar capability to MDCTP in the assessment of PBV and tumor vascularity [31,32,33,34]. In addition, the assessment of PBV could potentially aid in predicting tumor response [34,41,61]. The combination of anatomical and functional imaging highlights the potential of using dual-phase CBCT perfusion for treatment optimization and the prediction of the treatment response. However, two dual-phase CBCT acquisitions could potentially expose these patients to a high radiation dose. In addition, determining the value of this technique is difficult due to the long-term nature of treatment, which usually necessitates that patients undergo several sessions or different therapies over a long period of time.

This study has some limitations. First, the lack of consensus regarding the terminology for C-arm systems and related perfusion techniques made it difficult to devise a search strategy inclusive of all related articles. To mitigate any possible loss of articles, the search included cross-checking the references of the relevant literature and was expanded to include as many synonyms and terms as possible. Second, none of the studies included in this review assessed the relationship between the perfusion parameters and primary clinical outcomes. Third, the studies investigating 2D perfusion angiography incorporated small cohorts and demonstrated a large heterogeneity in the reference technique, the calculated perfusion parameters investigated, and the acquisition protocols. Fourth, the value of the studies in this review that investigated dual-phase CBCT perfusion could not be determined due to a lack of long-term follow-up. Last, the heterogeneity between the studies made it impossible to draw conclusions and pool the data.

For future studies investigating these techniques, we advise using the following naming scheme to avoid confusion and loss of information. The term flat-panel detector C-arm CT is used to describe any modern C-arm CT machine in angiography suites and hybrid operating rooms, considering all angiography suites now are equipped with C-arm systems incorporating flat-panel detector technology. The term 2D perfusion angiography is used to describe the 2D DSA perfusion imaging technique that relies on TDC analysis for the extraction of quantitative perfusion data from conventional 2D DSA images. The term dual-phase CBCT perfusion is used to describe a 3D technique for quantitative perfusion imaging with a flat-panel detector C-arm through acquiring two rotational volumes. For studies investigating 2D perfusion angiography, we recommend using a standardized acquisition protocol [40], including larger cohorts, and investigating the relationship with primary clinical outcomes. For studies investigating dual-phase CBCT perfusion, we recommend including larger, homogenous cohorts with long-term follow up.

## 5. Conclusions

In conclusion, the current review shows 2D perfusion angiography and dual-phase CBCT perfusion have potential for clinical implementation, adding new possibilities in treatment optimization. However, the selected literature is relatively scarce and heterogeneous. Our results show 2D perfusion angiography is feasible for monitoring endovascular therapy in patients with PAD but is currently limited by the lack of standardization of the acquisition protocol and extracted parameters. Dual-phase CBCT perfusion has shown potential for optimizing individualized therapy and the early detection of the treatment response; however, long-term follow-up data are lacking. Larger-scale randomized prospective studies focusing on clinical outcomes and standardization are required for the full understanding and clinical implementation of these techniques.

## Figures and Tables

**Table 1:** Study characteristics and overview of perfusion imaging techniques.

Study, Year, and Design	Cohort	Reference Technique	Variables Measured from C-Arm CT
Technique: 2D perfusion angiography			
Hinrichs 2016 [26], retrospective	PAD ^1^, n = 21	ABI ^2^	TTP ^3^, PD ^4^, and AUC ^5^
Ng 2019 [27], retrospective	PAD ^1^, n = 47	ABI ^2^, TBI ^6^	TTP ^3^, Imax ^7^, and washin ^8^ and washout ^9^ gradients
Lou 2016 [28], retrospective	PAD ^1^, n = 19	ABI ^2^	TTP ^3^
Su 2015 [29], prospective	PAD ^1^, n = 21	ABI ^2^, TcPO2 ^10^	TTP ^3^
Troisi 2021 [30], prospective	PAD ^1^, n = 24	TcPO2 ^10^	TDC ^11^
Technique: Dual-phase CBCT ^12^ perfusion			
Zhuang 2014 [31], prospective	HCC ^13^, n = 20	CTP ^14^ (CBV) ^15^	PBV ^16^
Peynircioğlu 2014 [32], prospective	HCC ^13^, n = 14	CTP ^14^ (CBV) ^15^	PBV ^16^
Rathmann 2017 [33], prospective	HCC ^13^, n = 16	CTP ^14^ (CBV) ^15^	PBV ^16^
Syha 2016 [34], retrospective	HCC ^13^, n = 25	CTP ^14^ (CBV) ^15^	PBV ^16^

^1^ peripheral arterial disease, ^2^ ankle–brachial index, ^3^ time to peak, ^4^ peak density, ^5^ area under the curve, ^6^ toe–brachial index, ^7^ maximum contrast intensity, ^8^ average contrast flow velocity before reaching maximum intensity, ^9^ average flow velocity from maximum contrast intensity till complete contrast washout, ^10^ transcutaneous oxygen pressure, ^11^ time–density curve, ^12^ cone-beam computed tomography, ^13^ hepatocellular carcinoma, ^14^ computed tomography perfusion, ^15^ cerebral blood volume, ^16^ parenchymal blood volume.

## Data Availability

This systematic review and the corresponding search strategy have been registered in the PROSPERO registry (http://www.crd.york.ac.uk/PROSPERO/, registration number: CRD42021243288).

## Appendix A

Search strategy PUBMED

(“Blood Circulation”[Mesh] OR blood circulat*[tiab] OR microcirculat*[tiab] OR micro-circulat*[tiab] OR micro-vascula*[tiab] OR microvascular*[tiab] OR macrocirculat*[tiab] OR macro-circulat*[tiab] OR “Regional Blood Flow”[Mesh] OR blood flow[tiab] OR “Perfusion”[Mesh] OR perfus*[tiab])

AND

(“Angiography, Digital Subtraction”[Mesh] OR digital subtraction angiograph*[tiab] OR DSA[tiab] OR rotational angiograph*[tiab] OR rotation angiograph*[tiab] OR cone beam[tiab] OR cone-beam [tiab] OR c-arm[tiab] OR c arm[tiab] OR CBCT[tiab] OR “Perfusion Imaging”[Mesh] OR FDCT[tiab] OR flat detector CT[tiab] OR flat panel detector CT[tiab])

AND

(“Ischemia”[Mesh] OR ischemi* [tiab] OR critical limb ischemia[tiab] OR critical ischemia[tiab] OR “Peripheral Arterial Disease”[Mesh] OR peripheral arterial disease[tiab] OR pad[tiab] OR peripheral vascular disease[tiab] OR pvd [tiab] OR “Abdominal Cavity”[Mesh] OR “Abdomen”[Mesh] OR abdom*[tiab] OR “Liver”[Mesh] OR liver*[tiab] OR “Splanchnic Circulation”[Mesh] OR splanchnic circul*[tiab] OR liver circul*[tiab] OR “Portal System”[Mesh] OR “Hepatocytes”[Mesh] OR hepat* [tiab] OR “Hepatic Veno-Occlusive Disease”[Mesh] OR “Hepatic Infarction”[Mesh] OR hepatic infarct*[tiab] OR “Hepatic Veins”[Mesh] OR hepatic vein*[tiab] OR “Hepatic Artery”[Mesh] OR hepatic arter*[tiab] OR “Kidney”[Mesh] OR kidn*[tiab] OR “Renal Circulation”[Mesh] OR renal circul*[tiab] OR “Renal Artery Obstruction”[Mesh] OR renal artery obstr*[tiab] OR “Renal Artery”[Mesh] OR renal arter*[tiab] OR “Renal Veins”[Mesh] OR renal vein*[tiab] OR “Spleen”[Mesh] OR spleen*[tiab] OR “Splenic Infarction”[Mesh] OR splenic infarc*[tiab] OR “Splenic Artery”[Mesh] OR splenic arter*[tiab] OR splenic vein*[tiab] OR “Prostate”[Mesh] OR prostat*[tiab] OR organ*[tiab])

NOT (“Heart”[Mesh])

NOT (“Animals”[Mesh] NOT “Humans”[Mesh])

Articles retrieved: 3022.

Search strategy EMBASE

(‘perfusion’/exp OR (‘blood circulat*’ OR microcirculat* OR ‘micro-circulat*’ OR ‘micro-vascula*’ OR microvascular* OR macrocirculat* OR ‘macro circulat*’ OR ‘blood flow’ OR perfus* ):ab,ti)

AND

(‘digital subtraction angiography’/exp OR (‘digital subtraction angiograph*’ OR DSA OR ‘rotational angiograph*’ OR ‘rotation angiograph*’ OR ‘cone beam’ OR ‘c arm’ OR CBCT):ab,ti)

AND

(‘vascular disease’/exp OR ‘peripheral vascular system’/exp OR ‘abdomen’/exp OR ‘liver’/exp OR ‘blood flow’/exp OR ‘circulation’/exp OR ‘portal system’/exp OR ‘liver cell’/exp OR ‘kidney’/exp OR ‘spleen’/exp ‘prostate’/exp OR (ischemi* OR ‘critical limb ischemia’ OR ‘critical ischemia’ OR ‘peripheral arterial disease’ OR pad OR ‘peripheral vascular disease’ OR pvd OR infarction* OR ‘abdominal cavit*’ OR abdom* OR liver* OR ‘splanchnic circul*’ OR ‘liver circul*’ OR hepat* OR ‘hepatic infarct*’ OR ‘hepatic vein*’ OR ‘hepatic arter*’ OR kidn* OR ‘renal circul*’ OR ‘renal artery obstr*’ OR ‘renal arter*’ OR ‘renal vein*’ OR spleen* OR ‘splenic infarc*’ OR ‘splenic arter*’ OR ‘splenic vein*’ OR prostat* OR organ*):ab,ti)

NOT (‘heart’/exp)

NOT (‘animal’/exp NOT ‘human’/exp)

Articles retrieved: 2511.

Search strategy CINAHL

(MM “Blood Circulation+” OR MM “Perfusion+” OR MH “Perfusion, Regional” OR MH “Tissue Perfusion” OR TI “blood circulat*” OR AB “blood circulat*” OR TI microcirculat* OR AB microcirculat* OR TI “micro-vascula*” OR AB “micro-vascula*” OR TI microvascular* OR AB microvascular* OR TI macrocirculat* OR AB macrocirculat* OR TI “macro-circulat*” OR AB “macro-circulat*” OR TI “blood flow” OR AB “blood flow” OR TI perfus* OR AB perfus*)

AND

(MH “Angiography, Digital Subtraction” OR MH “Perfusion Imaging” OR TI “digital subtraction angiograph*” OR AB “digital subtraction angiograph*” OR TI DSA OR AB DSA OR TI “rotational angiograph*” OR AB “rotational angiograph*” OR TI “rotation angiograph*” OR AB “rotation angiograph*” OR TI “cone-beam” OR AB “cone-beam” OR TI “c-arm” OR AB “c-arm” OR TI CBCT OR AB CBCT)

AND

(MM “Ischemia+” OR MM “Peripheral Vascular Diseases+” OR MM “Arterial Occlusive Diseases+” OR MM “Abdomen+” OR MH “Liver” OR MM “Splanchnic Circulation+” OR MM “Portal System+” OR MH “Hepatocytes” OR MH “Hepatic Artery” OR MH “Hepatic Veins” OR MM “Kidney+” OR MH “Renal Artery Obstruction” OR MH “Renal Circulation” OR MH “Renal Veins” OR MH “Splenic Vein” OR MH “Splenic Artery” OR MH “Spleen” OR MH “Renal Artery” OR MH “Prostate” OR TI ischemi* OR AB ischemi* OR TI “critical limb ischemia” OR AB “critical limb ischemia” OR TI “critical ischemia” OR AB “critical ischemia” OR TI “peripheral arterial disease” OR AB “peripheral arterial disease” OR TI pad OR AB pad OR TI “peripheral vascular disease” OR AB “peripheral vascular disease” OR TI pvd OR AB pvd OR TI infarction* OR AB infarction* OR TI “abdominal cavit*” OR AB “abdominal cavit*” OR TI abdom* OR AB abdom* OR TI liver* OR AB liver* OR TI “splanchnic circul*” OR AB “splanchnic circul*” OR TI “liver circul*” OR AB “liver circul*” OR TI hepat* OR AB hepat* OR TI “hepatic infarct*” OR AB “hepatic infarct*” OR TI “hepatic vein*” OR AB “hepatic vein*” OR TI “hepatic arter*” OR AB “hepatic arter*” OR TI kidn* OR AB kidn* OR TI “renal circul*” OR AB “renal circul*” OR TI “renal artery obstr*” OR AB “renal artery obstr*” OR TI “renal arter*” OR AB “renal arter*” OR TI “renal vein*” OR AB “renal vein*” OR TI spleen* OR AB spleen* OR TI “splenic infarc*” OR AB “splenic infarc*” OR TI “splenic arter*” OR AB “splenic arter*” OR TI “splenic vein*” OR AB “splenic vein*” OR TI prostat* OR AB prostat* OR TI organ*)

NOT (MH “Heart+”)

Articles retrieved: 78.

Search strategy COCHRANE

Blood circulat* OR microcirculat* OR micro-circulat* OR micro-vascula* OR microvascular* OR macrocirculat* OR macro-circulat* OR blood flow OR perfus*

AND

Digital subtraction angiograph* OR DSA OR rotational angiograph* OR rotation angiograph* OR cone beam OR cone-beam OR c-arm OR c arm OR CBCT

AND

Ischemi* OR critical limb ischemia OR critical ischemia OR peripheral arterial disease OR pad OR peripheral vascular disease OR pvd OR infarction* OR abdom* OR liver* OR splanchnic circul* OR liver circul* OR hepat* OR hepatic infarct* OR hepatic vein* OR hepatic arter* OR kidn* OR renal circul* OR renal artery obstr* OR renal arter* OR renal vein* OR spleen* OR splenic infarc* OR prostat* OR organ*

NOT “heart disease”

Articles retrieved: 554.
